# Retraction Note to: Cryptomycota: the missing link

**DOI:** 10.1007/s13238-014-0063-y

**Published:** 2014-06-14

**Authors:** Krishna Bolla, Elizabeth Jane Ashforth

**Affiliations:** Key Laboratory of Pathogenic Microbiology and Immunology, Institute of Microbiology, Chinese Academy of Sciences, Beijing, 100190 China

## Retraction Note to: Protein Cell 2012, 3(3): 161–162 DOI 10.1007/s13238-012-2013-x

This article has been retracted. Although the article “James, T.Y., and Berbee, M.L. (2011). No jacket required new fungal lineage defies dress code. Bioessays. doi:10.1002/bies.201100110” was cited, Bolla K unintentionally copied smaller parts of the text. The authors apologize to the authors of the BioEssays paper and the readers of *Protein and Cell*.

